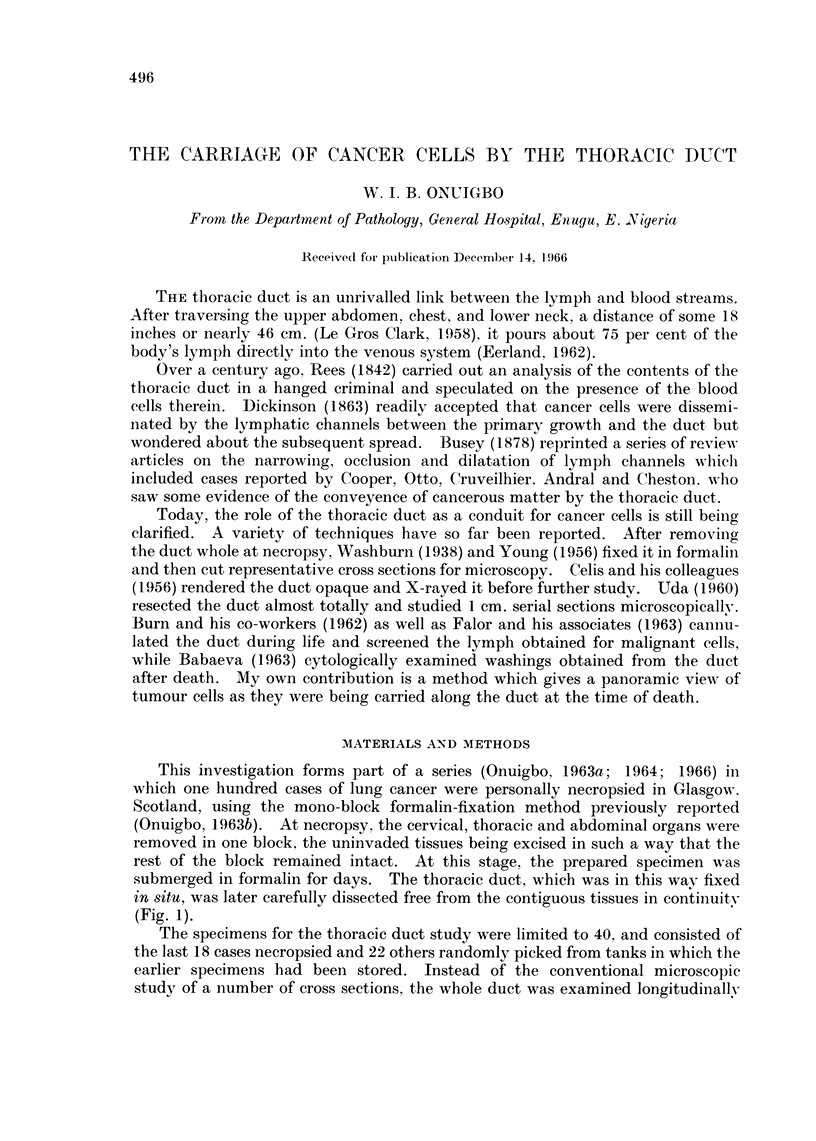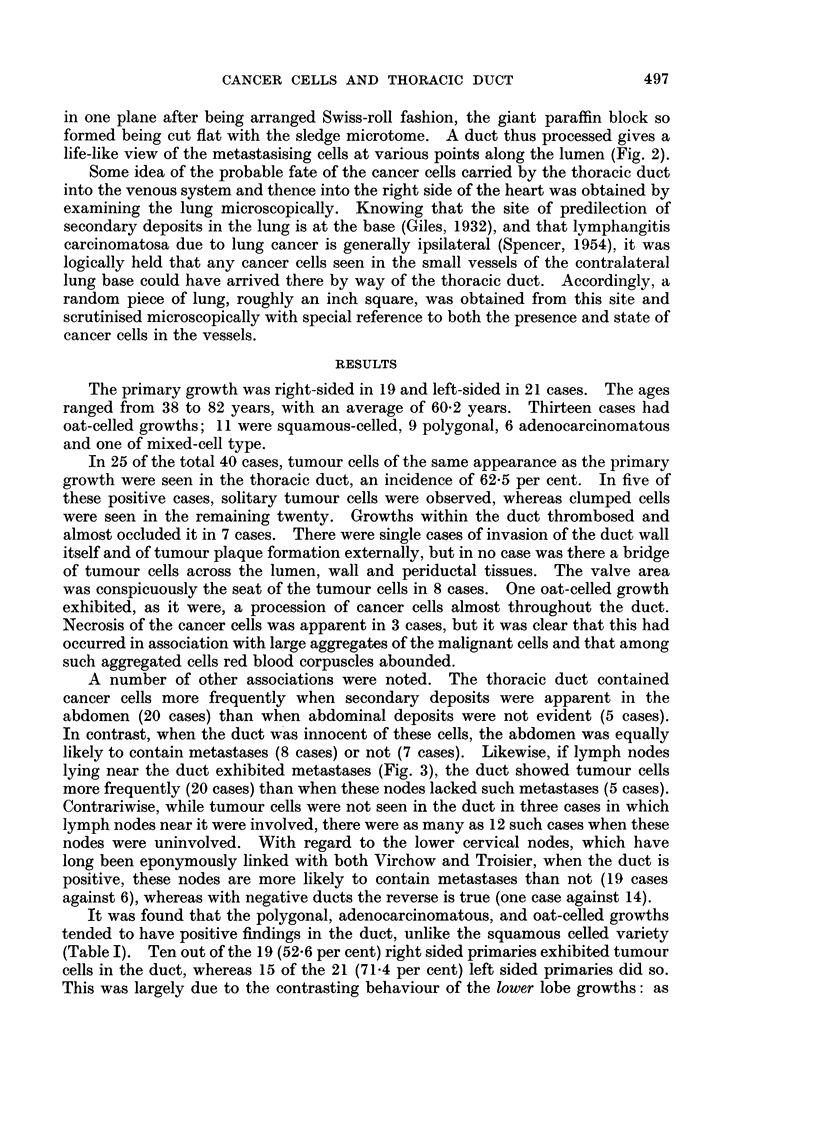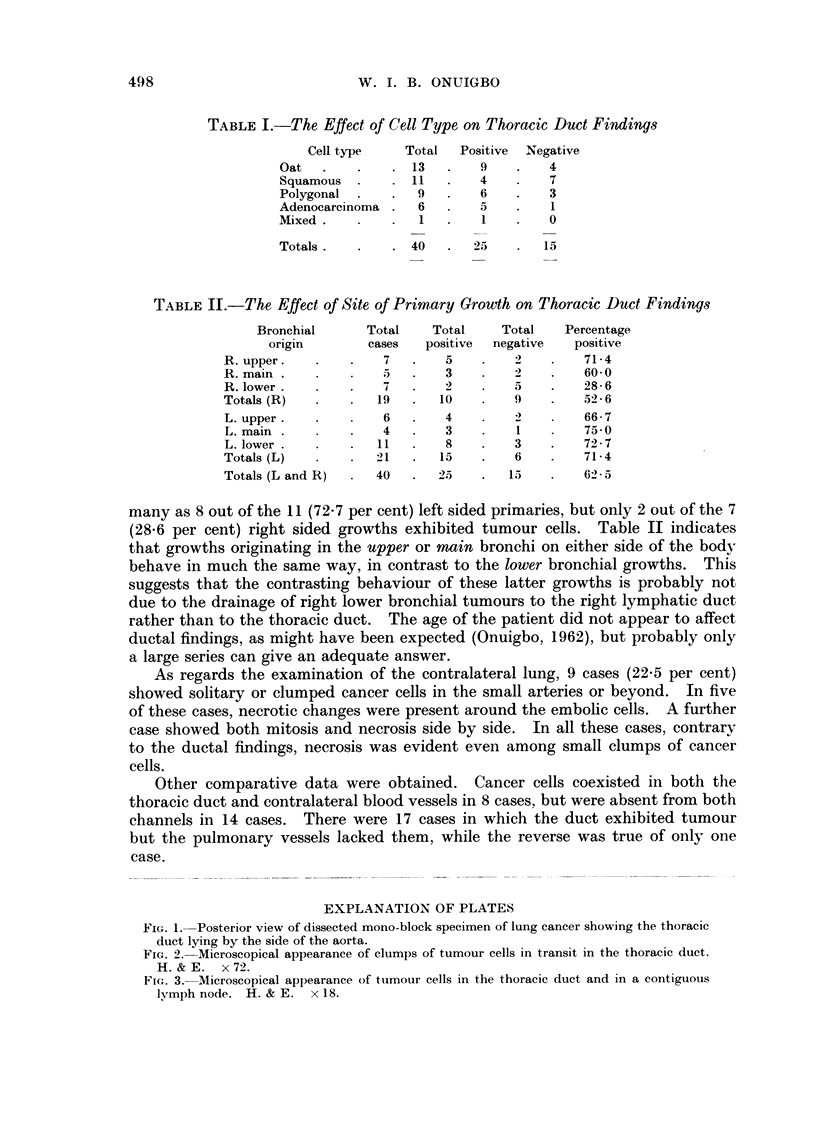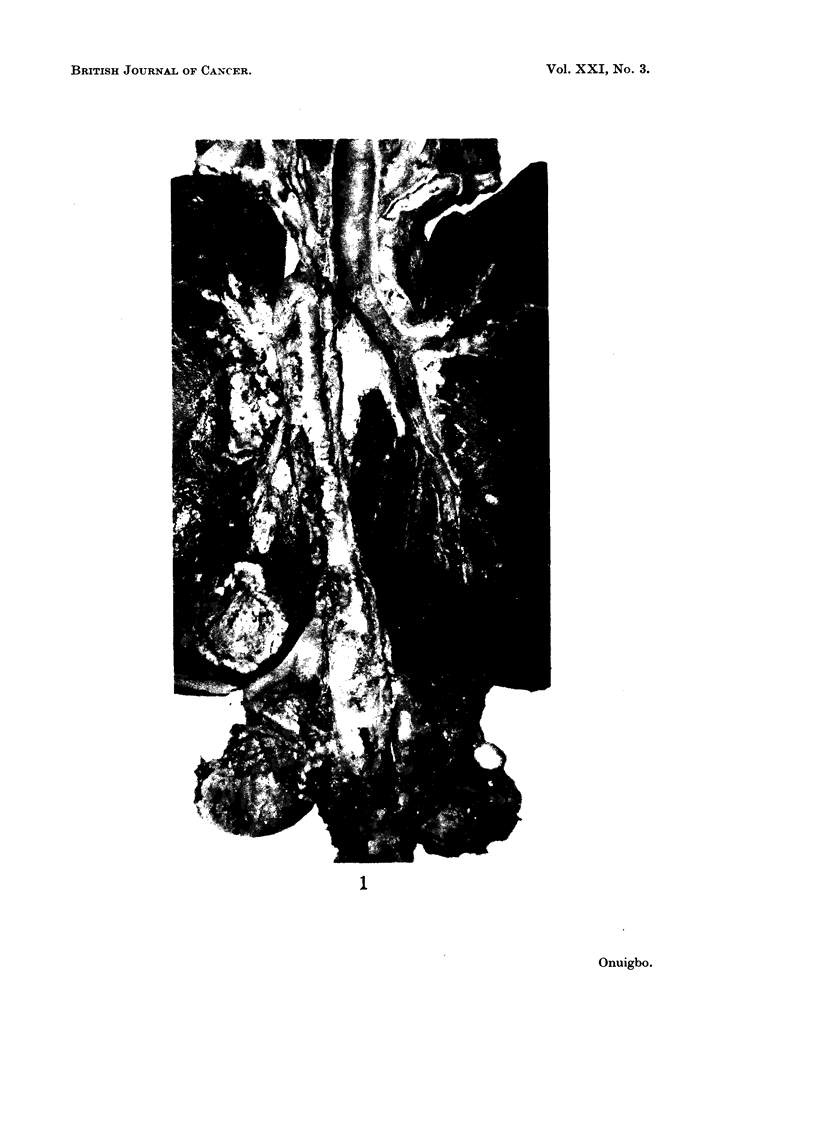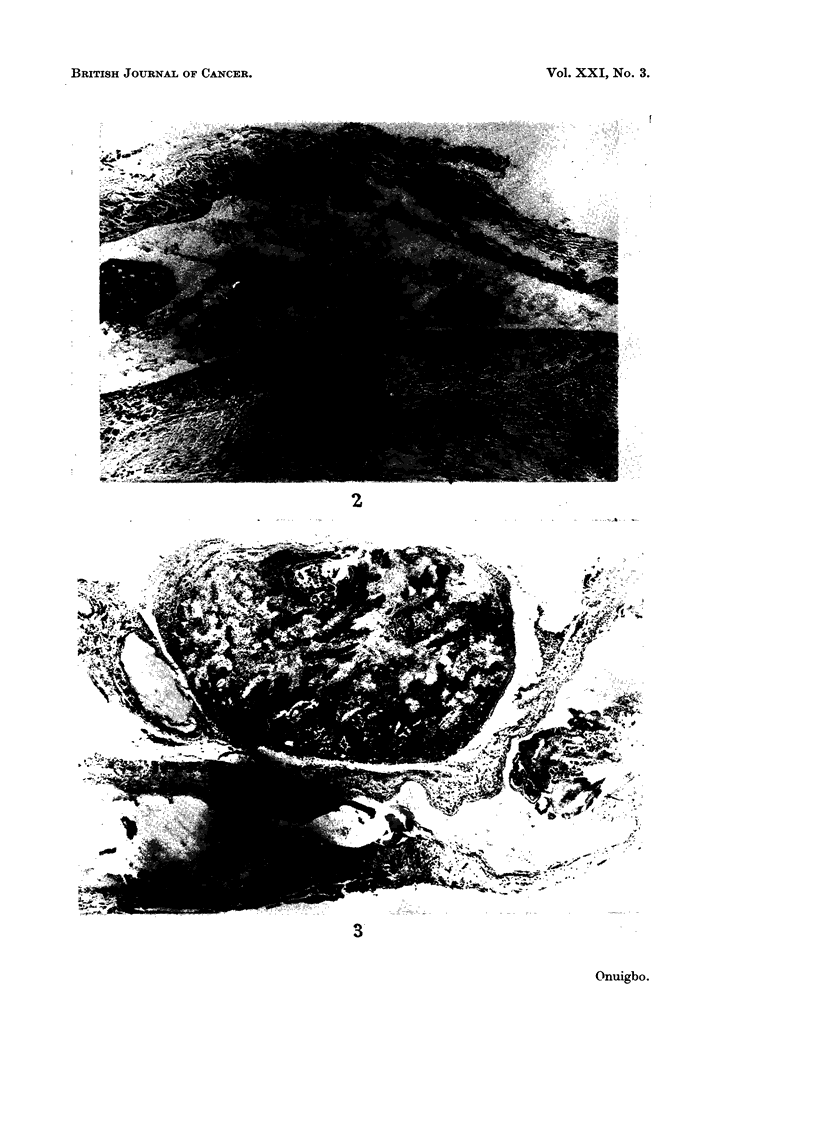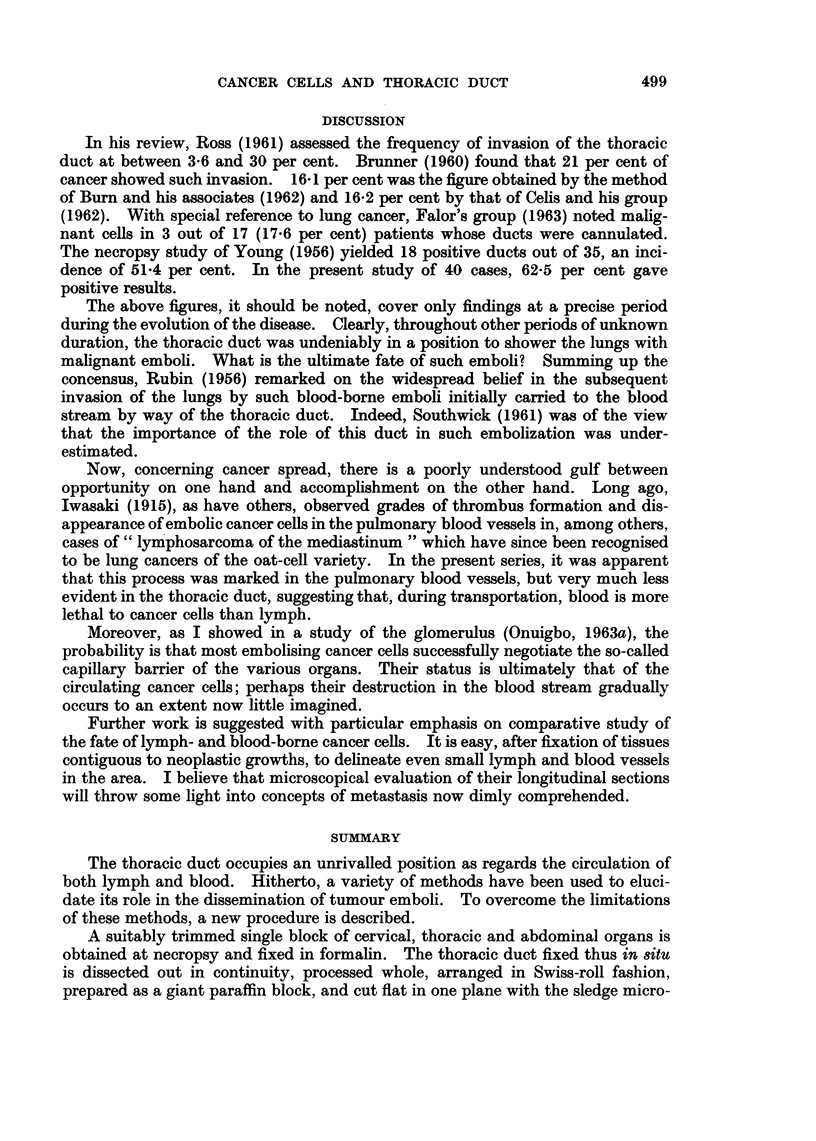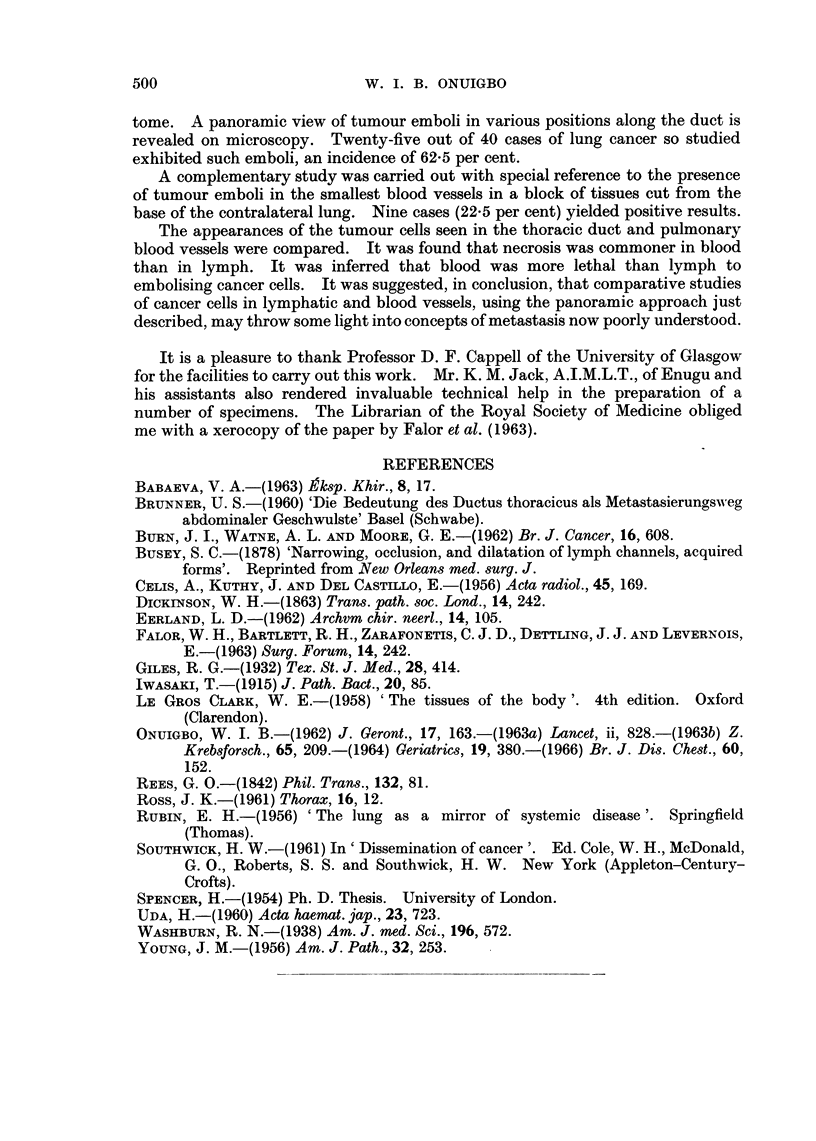# The carriage of cancer cells by the thoracic duct.

**DOI:** 10.1038/bjc.1967.57

**Published:** 1967-09

**Authors:** W. I. Onuigbo

## Abstract

**Images:**


### THE CARRIAGE OF CANCER CELLS BY THE THORACIC DUCT

W. I. B. ONUIGBO

*From the Department of Pathology, General Hospital, Enugu, E. Nigeria*

Received for publication December 14, 1966

THE thoracic duct is an unrivalled link between the lymph and blood streams. After traversing the upper abdomen, chest, and lower neck, a distance of some 18 inches or nearly 46 cm. (Le Gros Clark, 1958), it pours about 75 per cent of the body's lymph directly into the venous system (Eerland, 1962).

Over a century ago, Rees (1842) carried out an analysis of the contents of the thoracic duct in a hanged criminal and speculated on the presence of the blood cells therein. Dickinson (1863) readily accepted that cancer cells were disseminated by the lymphatic channels between the primary growth and the duct but wondered about the subsequent spread. Busey (1878) reprinted a series of review articles on the narrowing, occlusion and dilatation of lymph channels which included cases reported by Cooper, Otto, Cruveilhier, Andral and Cheston, who saw some evidence of the conveyence of cancerous matter by the thoracic duct.

Today, the role of the thoracic duct as a conduit for cancer cells is still being clarified. A variety of techniques have so far been reported. After removing the duct whole at necropsy, Washburn (1938) and Young (1956) fixed it in formalin and then cut representative cross sections for microscopy. Celis and his colleagues (1956) rendered the duct opaque and X-rayed it before further study. Uda (1960) resected the duct almost totally and studied 1 cm. serial sections microscopically. Burn and his co-workers (1962) as well as Falor and his associates (1963) cannulated the duct during life and screened the lymph obtained for malignant cells, while Babaeva (1963) cytologically examined washings obtained from the duct after death. My own contribution is a method which gives a panoramic view of tumour cells as they were being carried along the duct at the time of death.

### MATERIALS AND METHODS

This investigation forms part of a series (Onuigbo, 1963*a*; 1964; 1966) in which one hundred cases of lung cancer were personally necropsied in Glasgow, Scotland, using the mono-block formalin-fixation method previously reported (Onuigbo, 1963*b*). At necropsy, the cervical, thoracic and abdominal organs were removed in one block, the uninvaded tissues being excised in such a way that the rest of the block remained intact. At this stage, the prepared specimen was submerged in formalin for days. The thoracic duct, which was in this way fixed *in situ*, was later carefully dissected free from the contiguous tissues in continuity (Fig. 1).

The specimens for the thoracic duct study were limited to 40, and consisted of the last 18 cases necropsied and 22 others randomly picked from tanks in which the earlier specimens had been stored. Instead of the conventional microscopic study of a number of cross sections, the whole duct was examined longitudinally in one plane after being arranged Swiss-roll fashion, the giant paraffin block so formed being cut flat with the sledge microtome. A duct thus processed gives a life-like view of the metastasising cells at various points along the lumen (Fig. 2).

Some idea of the probable fate of the cancer cells carried by the thoracic duct into the venous system and thence into the right side of the heart was obtained by examining the lung microscopically. Knowing that the site of predilection of secondary deposits in the lung is at the base (Giles, 1932), and that lymphangitis carcinomatosa due to lung cancer is generally ipsilateral (Spencer, 1954), it was logically held that any cancer cells seen in the small vessels of the contralateral lung base could have arrived there by way of the thoracic duct. Accordingly, a random piece of lung, roughly an inch square, was obtained from this site and scrutinised microscopically with special reference to both the presence and state of cancer cells in the vessels.

### RESULTS

The primary growth was right-sided in 19 and left-sided in 21 cases. The ages ranged from 38 to 82 years, with an average of 60·2 years. Thirteen cases had oat-celled growths; 11 were squamous-celled, 9 polygonal, 6 adenocarcinomatous and one of mixed-cell type.

In 25 of the total 40 cases, tumour cells of the same appearance as the primary growth were seen in the thoracic duct, an incidence of 62·5 per cent. In five of these positive cases, solitary tumour cells were observed, whereas clumped cells were seen in the remaining twenty. Growths within the duct thrombosed and almost occluded it in 7 cases. There were single cases of invasion of the duct wall itself and of tumour plaque formation externally, but in no case was there a bridge of tumour cells across the lumen, wall and periductal tissues. The valve area was conspicuously the seat of the tumour cells in 8 cases. One oat-celled growth exhibited, as it were, a procession of cancer cells almost throughout the duct. Necrosis of the cancer cells was apparent in 3 cases, but it was clear that this had occurred in association with large aggregates of the malignant cells and that among such aggregated cells red blood corpuscles abounded.

A number of other associations were noted. The thoracic duct contained cancer cells more frequently when secondary deposits were apparent in the abdomen (20 cases) than when abdominal deposits were not evident (5 cases). In contrast, when the duct was innocent of these cells, the abdomen was equally likely to contain metastases (8 cases) or not (7 cases). Likewise, if lymph nodes lying near the duct exhibited metastases (Fig. 3), the duct showed tumour cells more frequently (20 cases) than when these nodes lacked such metastases (5 cases). Contrariwise, while tumour cells were not seen in the duct in three cases in which lymph nodes near it were involved, there were as many as 12 such cases when these nodes were uninvolved. With regard to the lower cervical nodes, which have long been eponymously linked with both Virchow and Troisier, when the duct is positive, these nodes are more likely to contain metastases than not (19 cases against 6), whereas with negative ducts the reverse is true (one case against 14).

It was found that the polygonal, adenocarcinomatous, and oat-celled growths tended to have positive findings in the duct, unlike the squamous celled variety (Table I). Ten out of the 19 (52·6 per cent) right sided primaries exhibited tumour cells in the duct, whereas 15 of the 21 (71·4 per cent) left sided primaries did so. This was largely due to the contrasting behaviour of the *lower* lobe growths: as

TABLE I.—*The Effect of Cell Type on Thoracic Duct Findings*

| Cell type | Total | Positive | Negative |
|---|---|---|---|
| Oat | 13 | 9 | 4 |
| Squamous | 11 | 4 | 7 |
| Polygonal | 9 | 6 | 3 |
| Adenocarcinoma | 6 | 5 | 1 |
| Mixed | 1 | 1 | 0 |
| Totals | 40 | 25 | 15 |

TABLE II.—*The Effect of Site of Primary Growth on Thoracic Duct Findings*

| Bronchial origin | Total cases | Total positive | Total negative | Percentage positive |
|---|---|---|---|---|
| R. upper | 7 | 5 | 2 | 71·4 |
| R. main | 5 | 3 | 2 | 60·0 |
| R. lower | 7 | 2 | 5 | 28·6 |
| Totals (R) | 19 | 10 | 9 | 52·6 |
| L. upper | 6 | 4 | 2 | 66·7 |
| L. main | 4 | 3 | 1 | 75·0 |
| L. lower | 11 | 8 | 3 | 72·7 |
| Totals (L) | 21 | 15 | 6 | 71·4 |
| Totals (L and R) | 40 | 25 | 15 | 62·5 |

many as 8 out of the 11 (72·7 per cent) left sided primaries, but only 2 out of the 7 (28·6 per cent) right sided growths exhibited tumour cells. Table II indicates that growths originating in the *upper* or *main* bronchi on either side of the body behave in much the same way, in contrast to the *lower* bronchial growths. This suggests that the contrasting behaviour of these latter growths is probably not due to the drainage of right lower bronchial tumours to the right lymphatic duct rather than to the thoracic duct. The age of the patient did not appear to affect ductal findings, as might have been expected (Onuigbo, 1962), but probably only a large series can give an adequate answer.

As regards the examination of the contralateral lung, 9 cases (22·5 per cent) showed solitary or clumped cancer cells in the small arteries or beyond. In five of these cases, necrotic changes were present around the embolic cells. A further case showed both mitosis and necrosis side by side. In all these cases, contrary to the ductal findings, necrosis was evident even among small clumps of cancer cells.

Other comparative data were obtained. Cancer cells coexisted in both the thoracic duct and contralateral blood vessels in 8 cases, but were absent from both channels in 14 cases. There were 17 cases in which the duct exhibited tumour but the pulmonary vessels lacked them, while the reverse was true of only one case.

### EXPLANATION OF PLATES

FIG. 1.—Posterior view of dissected mono-block specimen of lung cancer showing the thoracic duct lying by the side of the aorta.

FIG. 2.—Microscopical appearance of clumps of tumour cells in transit in the thoracic duct. H. & E. ×72.

FIG. 3.—Microscopical appearance of tumour cells in the thoracic duct and in a contiguous lymph node. H. & E. ×18.

1

Onuigbo.

2

3

Onuigbo.

### DISCUSSION

In his review, Ross (1961) assessed the frequency of invasion of the thoracic duct at between 3·6 and 30 per cent. Brunner (1960) found that 21 per cent of cancer showed such invasion. 16·1 per cent was the figure obtained by the method of Burn and his associates (1962) and 16·2 per cent by that of Celis and his group (1962). With special reference to lung cancer, Falor's group (1963) noted malignant cells in 3 out of 17 (17·6 per cent) patients whose ducts were cannulated. The necropsy study of Young (1956) yielded 18 positive ducts out of 35, an incidence of 51·4 per cent. In the present study of 40 cases, 62·5 per cent gave positive results.

The above figures, it should be noted, cover only findings at a precise period during the evolution of the disease. Clearly, throughout other periods of unknown duration, the thoracic duct was undeniably in a position to shower the lungs with malignant emboli. What is the ultimate fate of such emboli? Summing up the concensus, Rubin (1956) remarked on the widespread belief in the subsequent invasion of the lungs by such blood-borne emboli initially carried to the blood stream by way of the thoracic duct. Indeed, Southwick (1961) was of the view that the importance of the role of this duct in such embolization was underestimated.

Now, concerning cancer spread, there is a poorly understood gulf between opportunity on one hand and accomplishment on the other hand. Long ago, Iwasaki (1915), as have others, observed grades of thrombus formation and disappearance of embolic cancer cells in the pulmonary blood vessels in, among others, cases of " lymphosarcoma of the mediastinum " which have since been recognised to be lung cancers of the oat-cell variety. In the present series, it was apparent that this process was marked in the pulmonary blood vessels, but very much less evident in the thoracic duct, suggesting that, during transportation, blood is more lethal to cancer cells than lymph.

Moreover, as I showed in a study of the glomerulus (Onuigbo, 1963*a*), the probability is that most embolising cancer cells successfully negotiate the so-called capillary barrier of the various organs. Their status is ultimately that of the circulating cancer cells; perhaps their destruction in the blood stream gradually occurs to an extent now little imagined.

Further work is suggested with particular emphasis on comparative study of the fate of lymph- and blood-borne cancer cells. It is easy, after fixation of tissues contiguous to neoplastic growths, to delineate even small lymph and blood vessels in the area. I believe that microscopical evaluation of their longitudinal sections will throw some light into concepts of metastasis now dimly comprehended.

### SUMMARY

The thoracic duct occupies an unrivalled position as regards the circulation of both lymph and blood. Hitherto, a variety of methods have been used to elucidate its role in the dissemination of tumour emboli. To overcome the limitations of these methods, a new procedure is described.

A suitably trimmed single block of cervical, thoracic and abdominal organs is obtained at necropsy and fixed in formalin. The thoracic duct fixed thus *in situ* is dissected out in continuity, processed whole, arranged in Swiss-roll fashion, prepared as a giant paraffin block, and cut flat in one plane with the sledge microtome. A panoramic view of tumour emboli in various positions along the duct is revealed on microscopy. Twenty-five out of 40 cases of lung cancer so studied exhibited such emboli, an incidence of 62·5 per cent.

A complementary study was carried out with special reference to the presence of tumour emboli in the smallest blood vessels in a block of tissues cut from the base of the contralateral lung. Nine cases (22·5 per cent) yielded positive results.

The appearances of the tumour cells seen in the thoracic duct and pulmonary blood vessels were compared. It was found that necrosis was commoner in blood than in lymph. It was inferred that blood was more lethal than lymph to embolising cancer cells. It was suggested, in conclusion, that comparative studies of cancer cells in lymphatic and blood vessels, using the panoramic approach just described, may throw some light into concepts of metastasis now poorly understood.

It is a pleasure to thank Professor D. F. Cappell of the University of Glasgow for the facilities to carry out this work. Mr. K. M. Jack, A.I.M.L.T., of Enugu and his assistants also rendered invaluable technical help in the preparation of a number of specimens. The Librarian of the Royal Society of Medicine obliged me with a xerocopy of the paper by Falor *et al.* (1963).

### REFERENCES

BABAEVA, V. A.—(1963) *Éksp. Khir.*, **8**, 17.
BRUNNER, U. S.—(1960) 'Die Bedeutung des Ductus thoracicus als Metastasierungsweg abdominaler Geschwulste' Basel (Schwabe).
BURN, J. I., WATNE, A. L. AND MOORE, G. E.—(1962) *Br. J. Cancer*, **16**, 608.
BUSEY, S. C.—(1878) 'Narrowing, occlusion, and dilatation of lymph channels, acquired forms'. Reprinted from *New Orleans med. surg. J.*
CELIS, A., KUTHY, J. AND DEL CASTILLO, E.—(1956) *Acta radiol.*, **45**, 169.
DICKINSON, W. H.—(1863) *Trans. path. soc. Lond.*, **14**, 242.
EERLAND, L. D.—(1962) *Archvm chir. neerl.*, **14**, 105.
FALOR, W. H., BARTLETT, R. H., ZARAFONETIS, C. J. D., DETTLING, J. J. AND LEVERNOIS, E.—(1963) *Surg. Forum*, **14**, 242.
GILES, R. G.—(1932) *Tex. St. J. Med.*, **28**, 414.
IWASAKI, T.—(1915) *J. Path. Bact.*, **20**, 85.
LE GROS CLARK, W. E.—(1958) 'The tissues of the body'. 4th edition. Oxford (Clarendon).
ONUIGBO, W. I. B.—(1962) *J. Geront.*, **17**, 163.—(1963*a*) *Lancet*, ii, 828.—(1963*b*) *Z. Krebsforsch.*, **65**, 209.—(1964) *Geriatrics*, **19**, 380.—(1966) *Br. J. Dis. Chest.*, **60**, 152.
REES, G. O.—(1842) *Phil. Trans.*, **132**, 81.
ROSS, J. K.—(1961) *Thorax*, **16**, 12.
RUBIN, E. H.—(1956) 'The lung as a mirror of systemic disease'. Springfield (Thomas).
SOUTHWICK, H. W.—(1961) In 'Dissemination of cancer'. Ed. Cole, W. H., McDonald, G. O., Roberts, S. S. and Southwick, H. W. New York (Appleton-Century-Crofts).
SPENCER, H.—(1954) Ph. D. Thesis. University of London.
UDA, H.—(1960) *Acta haemat. jap.*, **23**, 723.
WASHBURN, R. N.—(1938) *Am. J. med. Sci.*, **196**, 572.
YOUNG, J. M.—(1956) *Am. J. Path.*, **32**, 253.